# Violaceous lunula with pigmented longitudinal ridge

**DOI:** 10.1016/j.jdcr.2023.11.018

**Published:** 2023-12-01

**Authors:** Rodolfo Valentini, Aziz Khan, Michael Murphy, Brett Sloan

**Affiliations:** a University of Connecticut School of Medicine, Farmington, Connecticut; b Department of Dermatology, UConn, Farmington, Connecticut

**Keywords:** cracked nail, glomus tumor, histopathology, immunohistochemistry, leiomyoma, longitudinal, nail tumor, spiradenoma, squamous cell carcinoma, subungual exostosis

A 73-year-old man presented with a 20-year history of a cracked, painful nail. The nail had been exquisitely sensitive to touch and cold temperatures. He denied systemic symptoms and personal history was only remarkable for cutaneous squamous cell carcinoma (SCC) on the left temple. Examination demonstrated a 5 mm, reddish-to-purple, triangular macule at the lunula, and a pigmented longitudinal ridge extending proximally from the distal nail plate edge (Fig 1).

**Fig 1:**
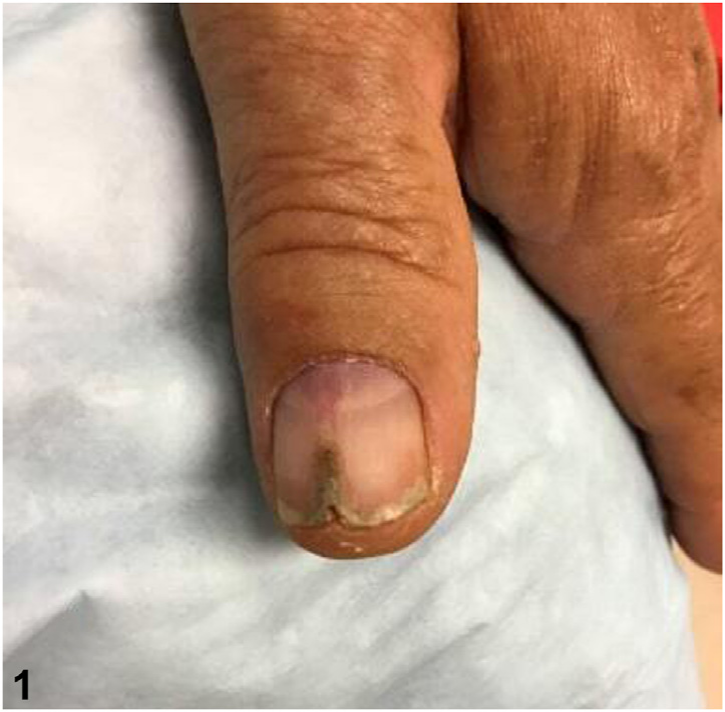

Punch biopsy of the nail matrix showed dermal proliferation of monomorphous, round, pink-blue cells surrounding delicate vascular spaces (Fig 2, *A-C*; varying magnifications). Immunohistochemistry (not shown) demonstrated diffuse, strong reactivity for smooth muscle actin (SMA) with focal/patchy positivity for desmin; AE1/AE3 (cytokeratin) was negative.

**Fig 2:**
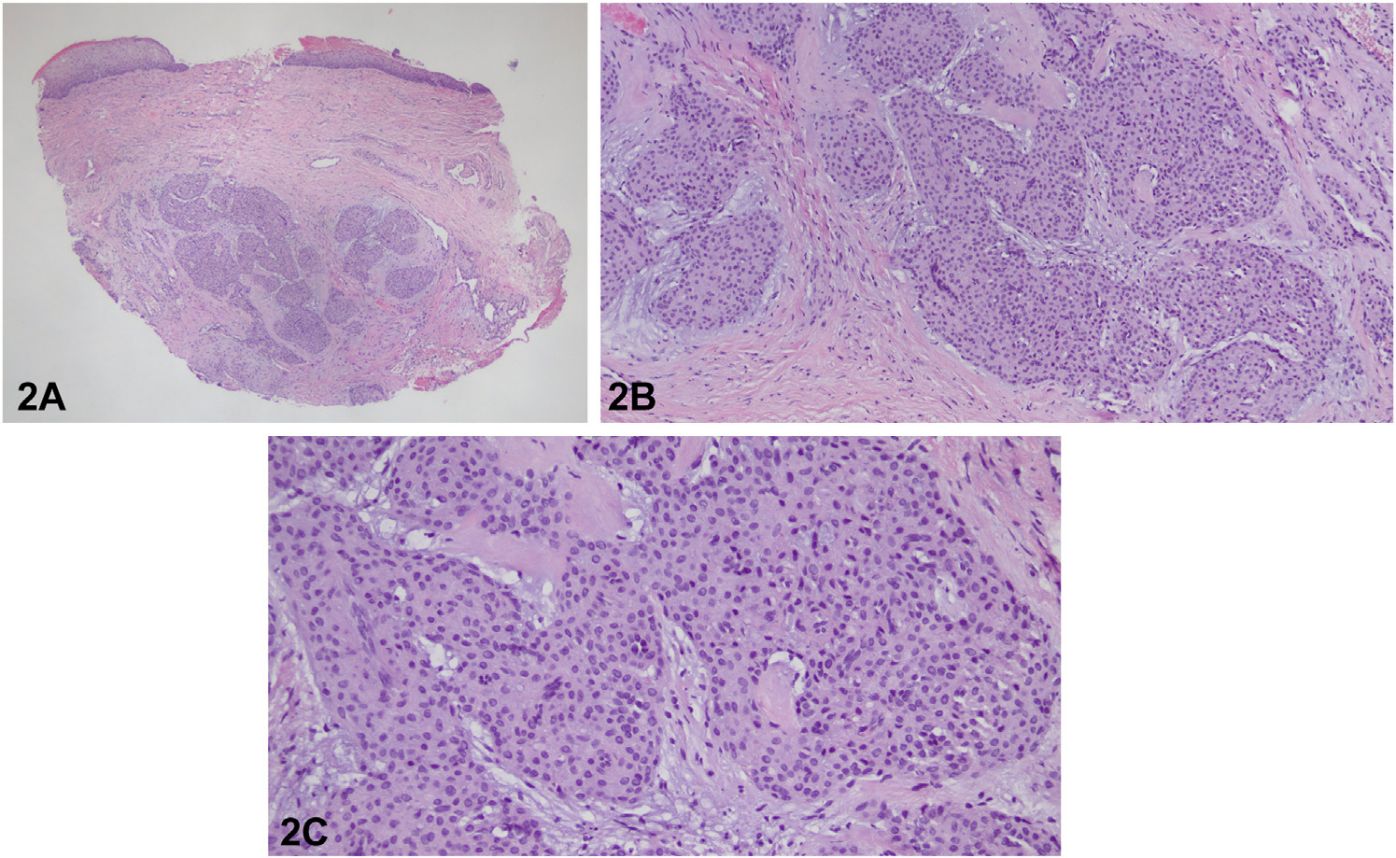

**Question 1: What is the most likely underlying diagnosis?**

A. Squamous cell carcinoma
B. Glomus tumor
C. Leiomyoma
D. Spiradenoma of the nail bed
E. Subungual exostosis

**Answers:**

A. Squamous cell carcinoma – Incorrect. SCC is the most common primary malignant neoplasm of the subungual region, mostly found in middle-aged Caucasian men.^1^ Its presence for 20 years without growth, extension, or malignant spread makes this less likely; however, one should always perform biopsy if suspicious. Histology reveals keratin pearls and nests of pleomorphic epithelial cells with abundant cytoplasm extending from the dermis to the epidermis.
B. Glomus tumor – Correct. Glomus tumors are benign vascular hamartomas common to the hand, but their small size may lead to a delay in diagnosis. Classic symptoms include a triad of cold hypersensitivity, undulating pain, and a well-defined pain site; longitudinal ridging, as seen here, may be infrequently associated with glomus tumors.^2^^,^^3^
C. Leiomyoma – Incorrect. Subungual leiomyoma is exceedingly rare, presenting in the 20’s and 30’s.^4^ Histopathology includes avascular, well-demarcated, fascicular, dermal proliferations of spindle cells with eosinophilic cytoplasm and cigar-shaped nuclei. IHC usuallydemonstrates strong positivity for both SMA and desmin.
D. Spiradenoma of the nail bed – Incorrect. Histopathology will present with well-demarcated, small, dark, peripheral cells and larger, pale, central cells arranged in trabeculae and tubules, with lymphocytes and stromal myxomatous changes. IHC may be positive for SMA, but not desmin in tumor-associated myoepithelial cells. CK7, CK8, and CK18 positivity are more consistent here.^5^
E. Subungual exostosis – Incorrect. Over 50% of cases present before 18 years of age, with toe involvement 80% of the time. Clinical features include a firm nodule that slowly grows over weeks to months. This patient did not report a growing nodule. Histology includes fibrocartilaginous tissue with a trabecular stalk.

**Question 2: What is the most appropriate next step in management for complete resolution?**

A. Surgical excision of the affected area
B. Antiviral suppression
C. Localized yeast injection
D. Liquid nitrogen treatment
E. Topical steroid application

**Answers:**

A. Surgical excision of affected area – Correct. Surgical excision is the recommended approach for complete resolution. A trans-ungual approach is most common, including the removal of the nail and excising the tumor.^2^ While allowing complete excision, this approach may not be cosmetically pleasing. Additionally, postsurgery recurrence is relatively common due to incomplete removal, development of a new lesion, or a missed satellite lesion.
B. Antiviral suppression – Incorrect. While effective for herpetic whitlow, viruses do not play a role in the etiopathogenesis of this condition.
C. Localized yeast injection – Incorrect. This is an effective treatment for verruca vulgaris but will not stimulate a sufficient immune response to eliminate the tumor.
D. Liquid nitrogen treatment – Incorrect. While liquid nitrogen causes local destruction, this is not recommended due to the tumor depth and imprecision.
E. Topical steroid application – Incorrect. This will have no effect on management and will likely be unable to penetrate the nail bed.

**Question 3: Which of the following is not useful in making a diagnosis?**

A. Hildreth’s test
B. Cold sensitivity test
C. Love’s pin test
D. Grattage Test
E. Clinical history taking

**Answers:**

A. Hildreth’s test – Incorrect. Hildreth’s test aids in diagnosing a glomus tumor as it utilizes the vascularity of this tumor to aid diagnosis.^2^ Hildreth’s test is performed by exsanguinating the affected extremity via elevation and torniquet followed by palpation of the affected area. A positive finding will show decreased sensitivity on exsanguination and sudden tumor site pain on re-perfusion.
B. Cold sensitivity test – Incorrect. Cold sensitivity test aids in diagnosing a glomus tumor as it is part of the classic clinical triad.^2^ The test is positive if the lesion becomes immensely painful on immersing the hand in cold water.
C. Love’s pin test – Incorrect. Love’s pin test aids in diagnosing a glomus tumor as it tests the pinpoint tenderness associated with the classic clinical triad. This test is performed by pressing the skin directly overlying the tumor with a pinhead, ballpoint pen, or paperclip end.^2^
D. Grattage Test – Correct. This is the name of test which is performed to elicit auspitz sign; removing scales from psoriasis lesions.
E. Clinical history taking – Incorrect. Clinical history can be immensely helpful in making a clinical diagnosis.

## Conflicts of interest

None disclosed.
